# Managing Risk or Supporting Desistance? A Longitudinal Study on the Nature and Perceptions of Parole Supervision in the Netherlands

**DOI:** 10.1007/s40865-018-0097-6

**Published:** 2018-12-05

**Authors:** Jennifer Doekhie, Esther van Ginneken, Anja Dirkzwager, Paul Nieuwbeerta

**Affiliations:** 1 Institute of Criminal Law and Criminology, Leiden University, Steenschuur 25, 2311 ES Leiden, The Netherlands; 2 Netherlands Institute for the Study of Crime and Law Inforcement, De Boelelaan 1077, 1081 HV Amsterdam, The Netherlands

**Keywords:** Parole, Desistance, Supervision style, Reentry, Risk management

## Abstract

**Purpose:**

Little is known about how ex-prisoners’ parole supervision experiences support or hinder the process of desistance. The aim of this article is to analyse the nature of parole supervision of Dutch (ex-)long-term prisoners in terms of official conditions, as well as the way in which parole officers (POs) and ex-prisoners navigate these conditions. The focus is particularly on the experienced supervision style and how this interacts with different dimensions of efforts at desistance.

**Methods:**

Twenty-three Dutch parolees were interviewed in depth at three waves starting in prison up to one year after their release from prison. A thematic analysis was undertaken to analyse the 69 interviews. In addition, the parole files of these ex-prisoners were examined containing information about conditions, violations and sanctions.

**Results:**

Parole files revealed the practice of highly engaged parole officers, who worked with parolees to strengthen factors known to foster desistance and tried to accommodate the difficulties of navigating ‘life outside’ after a relatively long prison sentence. However, the interviews showed that most parolees found their parole experience predominantly surveillance-oriented and not very helpful for desistance. Parole was experienced as most beneficial when parole officers were viewed as social workers or mentors and used their discretionary power to adjust conditions creating ‘space’ for trial-and-error.

**Conclusions:**

This longitudinal study suggests that a policy culture and discourse of risk management do not necessarily preclude desistance support in parole supervision in the Netherlands, due to discretionary power of parole officers.

**Electronic supplementary material:**

The online version of this article (10.1007/s40865-018-0097-6) contains supplementary material, which is available to authorized users.

## Introduction

Various scholars have advocated in favour of desistance-focused parole supervision [22, 53, 56]. Supervision should be aimed towards factors known to influence desistance: promoting a non-criminal lifestyle, strengthening pro-social bonds and ‘knifing off’ criminal networks, which could encourage individuals to move towards a non-criminal identity and a crime-free life [39, 48]. In the most ideal form, the parolee and the supervising officer work together to pinpoint a person’s strengths as well as the obstacles that could impede desistance [51].

However, many scholars argue that the current focus of parole in the USA and Europe is dominantly on crime-control and risk management instead of maintaining the original dual focus on rehabilitation and compliance [25, 57, 64]. This shift has been attributed to the ‘new penology’ [23], in which the social dimension has become less important and more weight has been given to supervising officers’ controlling tasks to monitor supervised individuals [25]. Supervisees are being perceived as individuals at risk who need to be closely monitored, while at the same time they are increasingly held responsible for their own change [77]. Such policies favouring more risk-based and surveillance approaches may contribute to attitudes that supervision can be ‘passed’ by simply ‘turning up’ and ‘signing in’ ([71], p.442). As a result, successful supervision outcomes (i.e. compliance, [9]) do not necessarily display real change.

Given this background, navigating between rehabilitation efforts and complying with more strict parole conditions seems a challenging task for parolees, especially when combined with the numerous re-entry challenges they face when leaving prison [28, 64]. The aim of this article is to analyse the nature of parole supervision of Dutch (ex-)long-term prisoners in terms of official conditions, as well as the way in which parole officers (POs) and ex-prisoners navigate these conditions. The focus is particularly on the supervision style (from PO and parolee perspective) and how this interacts with different dimensions of efforts at desistance. We used parole files to get an indication of the PO perspective and longitudinal interview data of a sample of 23 Dutch men, who were released after relatively long terms of imprisonment,^1^ to gain an understanding of the parolee perspective. The literature review, below, discusses the significance of parole supervision in relation to desistance, which is followed by a brief overview of the Dutch parole context.

### Prior Literature on Parole Supervision and Desistance

An increasing number of people are under some form of supervision in the community, a phenomenon that has been termed ‘mass supervision’ [55]. This includes people who are released on parole after serving a prison sentence. In 2016, the USA had approximately 870,000 individuals under parole supervision [37], England and Wales 70,650 and the Netherlands 1692 individuals [1]. This population is known to have complex needs in relation to, for example, housing, employment, mental health and substance abuse [22, 64]. Parole is aimed at reducing the risk of re-offending, but its relationship with desistance is complex and variable, and appears to partly depend on the relationship between parolees and their parole officers [32, 75].

Previous research on the experience of supervision has distinguished two different styles of supervision as perceived by individuals being subjected to it: a caseworker and a surveillance approach, oriented more towards rehabilitation and control, respectively [17, 27, 29, 69, 74]. While a caseworker approach focuses on assistance with problems and making efforts in order for supervised offenders to succeed in achieving goals, a surveillance approach is mainly aimed towards monitoring and crime-control. Supervising officers can also combine or switch between approaches if this is felt to be better suitable for the case in particular [17] or adjust their approach based on how they perceive the risk of re-offending [70]. Previous research on the experiences of supervision suggests that supervisees perceive a casework style as more beneficial to their process of change and that parole officers can facilitate offenders in their efforts to desist from crime [22]. Overall, supervisees appreciate the provision of practical help, moral support, and a good relationship with their PO, which includes consistency in seeing the same PO, being listened to, being recognised as an individual and being motivated and encouraged to solve problems on the road to desistance [33, 38, 41, 52, 68, 73, 75, 81, 83]. The impact of parole, therefore, may depend on building a reciprocal relationship between supervisees and their PO which heightens ‘commitment to desistance’ ([32], p. 388).

However, supervision can also be perceived as a more punitive experience for individuals subjected to it, which is particularly related to forms of intensive supervision (frequent check-ins and home visits) and the rise of ‘new surveillance’ technologies such as profiling, drug tests and electronic monitoring [46, 50]. Parole supervision in general, and intensive supervision in particular, can amplify the fear of being sent back to prison in case of violation after short and longer terms of imprisonment [42, 58]. Other examples of so-called pains of probation [19] are deprivation of time and autonomy in the case of frequent check-ins, being obligated as part of the conditions to share information about income, the threat of (re-)imprisonment, intrusive home visits, feelings of stigmatisation due to the ankle bracelet and feelings of isolation because of banning orders from certain places, people and situations [19, 31, 50, 59, 60, 63]. These aspects of supervision can make supervisees feel like they are being ‘processed’ or managed [41] and may hinder attempts at establishing a non-offender identity for those trying to desist. Moreover, these aspects could play a role in a ‘parolee performance’ of maintaining distance or deceiving their PO in order to simulate compliance [36].

Nonetheless, some research also suggests that surveillance-based practices such as curfews and electronic monitoring contribute to desistance by removing offenders from criminal networks or places, which decreases anti-social capital and creates opportunities to (re)connect with family and establish ties to legitimate employment [35, 79].

The present study seeks to further illuminate the way in which official parole conditions and the interaction between the parole officer and parolee are experienced in the context of different dimensions of desistance. It will be explored whether different types of conditions and supervision styles (caseworker and surveillance) can be distinguished and to what extent they are seen to contribute to desistance (or possibly, to offending).

Scholars use a variety of definitions to define desistance. For a prolonged period of time, desistance has been defined in terms of the absence of criminal behaviour or the cessation of offending in criminal career research [5, 13, 72]. In the past decades, desistance has also been studied as a complex process in which identity change seems to play an important role [26, 48, 62]. We are especially interested in parolees’ self-reported struggles and achievements related to supervision and the desistance process, and therefore examine supervision experiences in relation to the different dimensions of desistance as distinguished by Nugent and Schinkel [59]: act-desistance, identity desistance and relational desistance. Act-desistance refers to the plain state of non-offending (no recidivism) while identity desistance includes a shift to embracing a pro-social identity, such as a family man, a good parent or a ‘worker’ [40, 61] which helps individuals to move away from their identity as an offender. ‘Hooks for change’ such as employment and family can serve as an avenue to craft these (new) roles or identities as they provide meaning, a sense of purpose and an opportunity to present a changed self [26, 48, 61]. Finally, relational desistance denotes another important dimension of desistance: others recognising a person’s change.^2^ Note that these dimensions of desistance are not mutually exclusive and are not necessarily ordered in a specific way.

Previous research in this area has tended to rely on parolees who were invited by their POs to participate in the study, were committed to desist, eventually desisted or were seen as successful in their supervision endeavours (see for example [31, 33, 38, 41, 68, 73]). Therefore, current knowledge is primarily based on selective and more successful samples of parolees/probationers. A strength of the present study is that men were approached while in prison, and, therefore, included both men who were committed to desist and eventually desisted, and men who were not committed to desist and who continued with crime. Furthermore, by analysing parole files in addition to qualitative interview data, we were able to compare the official case files, which document the PO’s perspective, with the experiences of parolees. It is also important to consider the national context in which the research was conducted. Compared with the USA, the Netherlands is known for having relatively short-term sentences, one of the lowest prison populations in Europe with 53 prisoners per 100,000 inhabitants [2] and a more developed welfare system. Nevertheless, also in the Dutch mild penal climate, it is possible to identify a trend towards greater surveillance of the population of ex-prisoners [6].

### ‘To a safer society’: Parole in the Netherlands

In the Netherlands, the Public Prosecution Service (OM) officially imposes the conditions tied to prisoners’ release^3^; then, the Dutch Prison Service gives the task of the actual supervision and support of these parolees to the Probation Service (*Reclassering*) [7, 24]. The Probation Service professionalised its supervision task in 2010 with a project named ‘Redesign Supervision’ (*Redesign Toezicht*) which was focused on two main tasks: surveillance (control) and support [6]. However, surveillance tasks for parole officers are described in much more detail than support, and findings from an evaluation study of this project pointed out that more attention should be paid to clear descriptions of supporting tasks and instructions on how to combine these tasks [66]. It was also suggested that supervision could be more effective if the role of the parole officer as a ‘broker’ could be expanded to a ‘change agent’, emphasising the impact of the relationship between the parolee and PO.

Each year, approximately 1000 individuals are conditionally released which is 2.5% of all released prisoners [14]. This low percentage is mostly due to the fact that in the Netherlands, only prisoners with a minimum sentence of 1 year are eligible for conditional release. The average number of specific conditions tied to release went up from 2.5 in 2012 to 3.5 in 2016 and these were imposed in 70% of the cases [14]. The number of *requests* for revocations as a result of violating conditions more than doubled from 95 in 2012 to 211 in 2013 and then decreased to 193 in 2017 [7, 67]. The number of *actual* revocations (full or partial) appeared to have slightly increased in the period 2012–2014. While formal policy stipulates that violations of conditions are followed by an official response, little is known yet about how parole officers respond to violations and to what extent their discretionary power shapes informal reactions to violations [7].

## Methodology

This paper aims to analyse the nature of parole supervision in the Netherlands and focuses on the supervision style and how parolees and POs navigate the conditions of their release during the transition from prison to society. Findings of the current study are therefore based on two primary data sources to include different perspectives: (a) 69 interviews were carried out as a sub-study of the Prison Project to get an insight into parolees’ experiences and (b) parole case files were analysed for the PO perspective. In addition, criminal records were consulted to explore if self-reported offending came to the attention of the criminal justice system. Data triangulation in parole research can be of added value in unravelling inconsistencies and offering additional understanding from another data source [3].

### Interview Data Prison Project

As part of a larger endeavour to study the consequences of imprisonment, longitudinal interview data were collected over three waves to gain insight into the subjective experience of supervision in a Dutch sample of (ex-)prisoners. The larger Prison Project was approved by an Ethical Commission and targeted men, born in the Netherlands and aged 18–65 [16]. In addition to these criteria, this project also focused on men who (a) were imprisoned for a—to Dutch standards—relatively long time, i.e. between 2 and 4 years at the moment of release,^4^ (b) were convicted for a criminal offence (not on appeal), (c) were not in a facility for ‘revolving door’ offenders, detained under hospital order or in a minimum security prison, and (d) were not convicted for a sex offence (see also [18]).^5^ The Dutch Prison service provided a list of all men across the country fitting the eligibility criteria with already set (expected) release dates in the period September 2014–September 2016. At that time, the list contained 84 men and during the first interview wave in October 2015, 44 were approached in prison in person by the first author. In a separate room where no staff members were present, they were informed about the study and received an information leaflet. Also, it was clarified that participation was voluntary and the decision to participate or not would not have any consequences for their sentence. After ensuring confidentiality, most prisoners (*n* = 36) agreed to participate and the (on average) 1.5-h interview was held individually in a private room. Eight interviews were excluded afterwards because they did not meet the inclusion criteria after all,^6^ which resulted in 28 participants in the first wave (T1). Three months (T2) and a year after release (T3), all men were located using contact information gathered at the previous interview(s) or with help from the Dutch Probation Service. In total, 23 men consented to participate in both follow-up interviews resulting in a total of 69 interviews.^7^ Locations of both post-release interviews were by default at the parolee’s home or the assisted living facilities where they were staying (30%), unless they preferred to be interviewed at an alternative location including public areas (37%) and private rooms at the supervision office (13%). Some interviews were carried out in prison if they had returned (20%). All interviews were conducted by the first author of this paper and as a token of gratitude, participants received €10 cash after completing the interview. The men were on average 27 years of age (range 21–53) and had been serving sentences between 30 and 66 months. The average time spent in prison at the time of release was 38 months. The average anticipated length of supervision upon release was 20 months (range 12–26 months). Table 1 presents some descriptives of the sample.

**Table 1:** Descriptives of parolees in this study (*N* = 23)

Alias	Age	Offence type	Partner T1	Partner T2	Partner T3	Employment T2^1^	Employment T3
Ab	25–29	Robbery	Yes	Yes	Yes	No	No
Casper	35–39	Kidnapping, extortion	No	No	No	No	No
Dave	20–24	Robbery	No	No	No	Formal	Formal
Leon	20–24	Robbery	No	No	Yes	No	No
Peter	50–54	Fraud	Yes	Yes	No	Formal	Formal
Tom	30–34	Robbery	Yes	Yes	No	Formal	No
Tony	20–24	Robbery	No	No	No	No	Informal and illegal
Bart	20–24	Robbery	Yes	No	No	No	No
Chris	25–29	Robbery	No	No	Yes	No	Formal
Isaac	30–34	Robbery	No	No	No	No	No
Jack	25–29	Robbery	No	No	No	Informal	Informal
Martin	20–24	Robbery	Yes	No	No	No	No
Milo	25–29	Attempted manslaughter	No	Yes	Yes	Formal	Formal
Nathan	20–24	Robbery	No	No	No	Formal	Formal
Oscar	20–24	Robbery	No	No	Yes	No	No
Pascal	30–34	Robbery	No	No	Yes	No	Formal
Roy	25–29	Robbery	Yes	Yes	No	No	No
Rudy	30–34	Robbery	No	No	No	No	No
Sam	20–24	Robbery	No	Yes	Yes	Education	Formal
Simon	20–24	Robbery	Yes	Yes	No	Formal	No
Vince	25–29	Burglary	No	No	Yes	Informal	Informal
Wessel	20–24	Attempted manslaughter	No	No	No	No	No
Xavier	20–24	Robbery	No	No	No	Education	Education

^1^Working outside the formal economy, but not engaged in activities violating criminal law, was referred to as informal employment

Although the semi-structured interview protocol included a broad range of topics, including the experience of imprisonment, motivation to desist and re-entry challenges, for this paper, we were interested in how participants experienced their supervision and relationship with their PO, and additional conditions, such as the curfews, location bans, orders to stay away from certain people (victims, co-offenders) and the use of electronic monitoring. The current analysis was focused on questions such as ‘How do you feel about being supervised in your conditional release period?’ asking about their experiences with complying with the rules, but also if they felt assisted by their PO in the process. These topics were present in all three interview waves (even in the first in-prison interview concerning expectations regarding supervision) and allowed us to gain insight into the lived experiences of supervision during the first year after release.

**Fig. 1:**
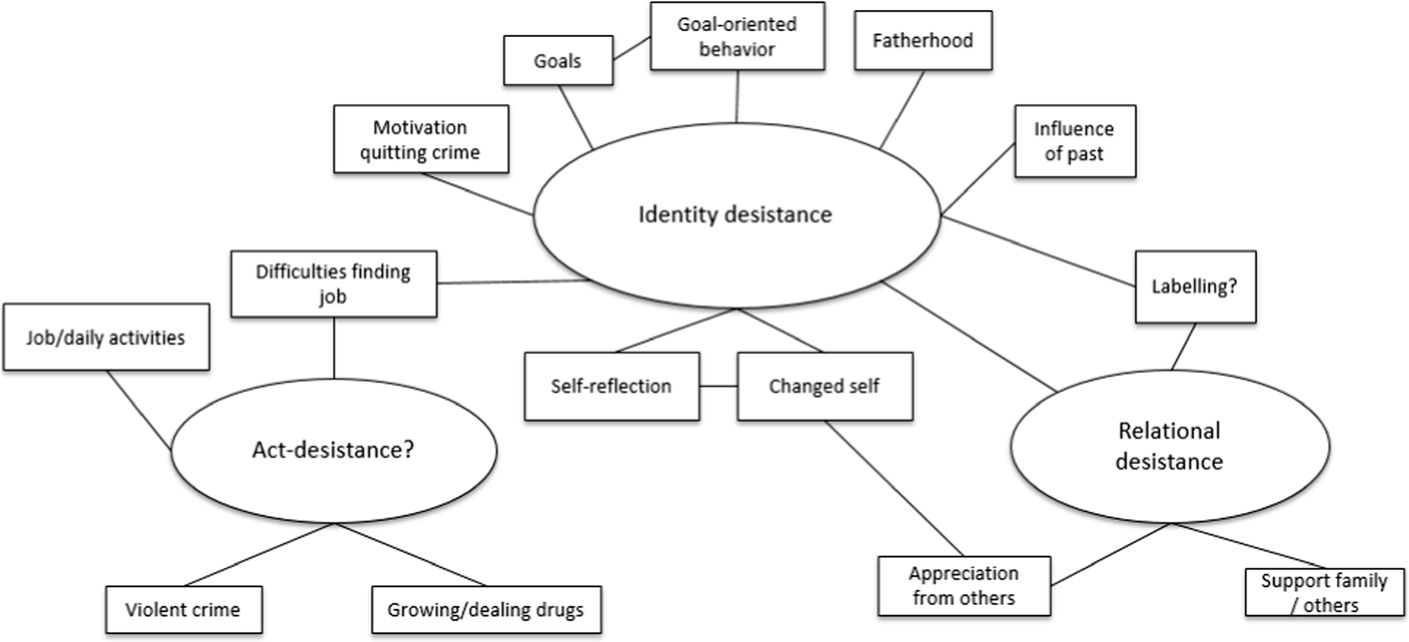
Thematic map showing the used codes of three interview waves to identify different aspects of desistance

A thematic analysis was undertaken to analyse the longitudinal data [10, 12]. The interviews of all three waves (a total of 69 interviews) were given codes of all topics concerning re-entry and desistance. Atlas.ti facilitated the process of data management and analysis. Figure 1 displays the thematic map of codes used across three waves, showing how the different dimensions of desistance were interpreted. For example, we explored identity desistance with fragments about, for example, *trying to be* a non-criminal, a non-drug user, a family man (good father, caring partner), a son or a ‘worker’ [40, 61]. We looked not only for the intention or desire to connect to such roles,but also for actively taking steps towards a pro-social identity, for example by signing up for drug treatment voluntarily, attending parent teacher meetings at school, going to job interviews or even cooking regularly at home for parents. When participants mentioned receiving support and appreciation from parents, partner, friends or from the parole officer in their efforts to go straight or how they fulfilled a new role, we identified this as relational desistance. In line with Nugent & Schinkel [59], we did not assume a temporal ordering of the different dimensions.

To examine act-desistance, all participants were given a pseudonym to identify quotes and in addition also a label consisting of three letters (A, C, N) in different combinations. These labels refer to the act-desistance (self-reported and official non-offending) from pre-release up to a year after release. The first letter was derived from the pre-release interview, where participants’ expectations for future criminal behaviour were classified into criminal (C), meaning continuing crime; non-criminal (N), meaning refraining from crime; or ambivalent (A), meaning unsure about continuing or refraining from crime. At the two follow-up interviews, behaviour was classified as criminal (C) or non-criminal (N).^8^ When no criminal activities were reported and no official offending could be found on criminal records in the period of the two follow-up interviews, we categorised participants as ‘desisters’ (*n* = 14) (combinations NNN, ANN and CNN) and as ‘persisters’ (*n* = 9) when involvement in crime was self-reported in at least one of the two post-release interviews (combinations NCC, CCC, ACC, NNC and CCN).^9^ Official offending in criminal records corresponded to self-reported offending, but contained less (or less serious) offending than what was self-reported by participants. We consulted the criminal records for the purpose of triangulation of the theoretical construct of act-desistance. This also allowed to support certain findings, for example, that some parolees were indeed involved in a form of ‘gameplaying’ [11], as will be discussed later in this article.

### Parole Case Files

The 23 parole files of the men in our sample were examined. These files contained information from the Prosecutorial Office about the imposed specific conditions, such as check-ins, participation in courses and electronic monitoring. In addition, information about violations and sanctions up to a year after conditional release were examined. Aside from data about missed check-ins and official warnings, parole files also offered us insight into the practice of POs since almost all of them reported extensively about their contact with the parolees: doubts with regard to a parolee’s rehabilitation efforts, contemplations concerning tolerance for missteps and considerations whether or not to sanction violations.

## Findings

### Conditions of Release: Surveillance or Rehabilitation?

In this section, first the findings from examining the parole case files will be presented. Initially, 87% of the sample was placed at the most intensive level (high-risk) of supervision which entailed weekly check-ins. The frequency of check-ins decreased for all participants who were still under supervision a year after release.

Table 2 presents information from the case files on (the co-occurrence of) parolees’ specific release conditions, sorted by the total number of conditions. Mandatory check-ins were part of the specific conditions in all cases (*n* = 23). Participants also had to comply with other controlling conditions such as home confinement with curfews (*n* = 13) and location bans, sometimes for multiple cities (*n* = 11), both enforced by electronic monitoring. Although the imposed specific conditions revealed a focus on surveillance and monitoring, importance was also given to more rehabilitative conditions. Almost two thirds of the sample were ordered to undergo psychological treatment (*n* = 14), which usually involved an assessment to determine if someone needed psychological help and in case they did, the provision of treatment. Various participants had to live in an assisted living facility as part of their reintegration (*n* = 10), take part in behavioural or vocational courses (*n* = 7) and a few had to undergo drug treatment (*n* = 4). Surveillance and rehabilitative conditions were combined in almost all cases. Only two parolees had to comply exclusively with surveillance conditions and the far majority (83%) was subjected to three or more specific conditions.

**Table 2:** Specific release conditions of parolees in this study (sorted by the total number of conditions), their perceptions of the parole experience at T2 and T3 and their desistance trajectories (*N* = 23)

Alias	Check-ins	PT	HC	LB	ALF	DB	No contact order	Courses	DT	Other	Perception of parole by parolees T2	Perception of parole by parolees T3	Trajectory
Casper	x							x			Casework	Casework	ANN
Milo	x						x				Surveillance	Surveillance	CNN
Roy	x		x								Surveillance	Surveillance	CCC
Tom	x	x									Casework	Casework	NNC
Ab	x	x	x								Surveillance	In prison	CCC
Dave	x	x			x						Surveillance	Surveillance	NNN
Peter	x	x	x								Casework	No supervision	NNN
Simon	x				x					x	Surveillance	Surveillance	NNN
Tony	x	x						x			Surveillance	Surveillance	CCC
Nathan	x		x					x		x	Surveillance	Surveillance	NNN
Sam	x	x	x	x							Surveillance	Surveillance	NNN
Vince	x					x		x	x		Casework	Casework	ANN
Bart	x		x	x			x	x			Surveillance (P)	In prison	NCC
Pascal	x		x	x		x			x^1^		Surveillance	Casework	CNN
Rudy	x			x	x	x	x				Surveillance	Surveillance	CCN
Xavier	x	x			x	x		x			Casework	Casework	NNN
Wessel	x	x	x	x	x	x					Casework (P)	In prison	CCC
Chris	x	x	x	x	x					x	Casework	Casework	ANN
Martin	x	x	x	x			x			x	Surveillance	Surveillance (P)	ACC
Isaac	x	x	x	x	x	x		x			Casework	Casework	ANN
Jack	x	x	x	x	x	x			x		Surveillance (P)	Surveillance	NNN^2^
Leon	x	x		x	x	x	x			x	Surveillance (P)	Surveillance	CCC
Oscar	x	x	x	x	x		x		x	x	Casework	Casework	NNN

*PT*, psychological treatment; *HC*, home confinement (with curfews and electronic monitoring); *LB*, location ban (with electronic monitoring); *ALF*, assisted living facility (sometimes with additional curfews); *DB*, drug ban/drug tests; *DT*, drug treatment. (P), in prison at the time of the interview. When someone was back in prison at the time of the interview, they were asked about their experiences with parole supervision after release until they were imprisoned again

The labels of the trajectories refer to the act-desistance (self-reported and official non-offending) from pre-release up to a year after release. The first letter was derived from the pre-release interview, where participants’ expectations for future criminal behaviour were classified into criminal (C), meaning continuing crime; non-criminal (N), meaning refraining from crime; or ambivalent (A), meaning unsure about continuing or refraining from crime. At the two follow-up interviews, behaviour was classified as criminal (C) or non-criminal (N)

^1^Pascal only had to participate in drug treatment if he violated the specific condition of the drug ban

^2^Three months after release, Jack was in prison again for violating his licence conditions, because he had no official registration address which was needed for the conditional release. We classified him as non-criminal (N) at all three waves, even though he was in prison at the time of the second interview

With regard to violations of the conditions, ten men missed at least one, but usually multiple check-ins, eight men ignored a curfew at least once and five had at least one positive drug test (mostly for cannabis) or relapse.^10^ Not all violations were sanctioned. Responses to violating specific conditions mostly consisted of an official warning or a reprimand,^11^ while revocations were almost exclusively requested when a new crime was committed. A total of 30 sanctions (of 14 participants) could be found in the parole files: almost half of the sanctions were official warnings, a quarter concerned revocations of conditional release and a few were reprimands. A typical reason to give an official warning was to stress the importance of following the rules and allow for second (or final) chances if the parolee was perceived to be motivated and to have good intentions (see also [4]). The following note of a PO is a good illustration of the deliberation of such a response:

Given the motivation he shows to get his life back on track, the Probation Service advices to give Mr. [name parolee] an official warning which serves as a second chance in order to successfully finish his supervision and the programme at [name reintegration organization]. (Note from parole file Nathan)

Alternative responses from POs to unsanctioned violations included having a serious talk with the parolee and denying a request for minimising curfew hours in order to gain more freedom.

It is worth mentioning that, despite supervision literature pointing out a shift towards a surveillance approach (which is also reflected in the imposed conditions), the PO’s *practice* in the far majority of the cases reflected a caseworker approach. Sometimes, this turned into a surveillance approach focused on monitoring and sustaining compliance when the parolee seemed reluctant to cooperate (see [47]). Yet in general, POs mediated in problematic family situations, showed understanding for the impact of imprisonment and requested to slightly adjust curfew hours or location bans if they were thought to hinder reintegration opportunities. Parole files indicated that issues such as parolees getting used to freedom and practicing with aspects of pro-social life (such as taking a date out for dinner or visiting children in the restricted area) were seen as valuable by POs and were reasons to use their discretionary power to adjust conditions. Examples of POs calling credit bureaus to manage debts and assisting with administrative matters were also found. Furthermore, POs ‘defended’ parolees when they were being subjected to criticism from external organisations, such as job agencies. Illustrations of the above can be found in the following notes from POs found in the parole files:

I agree that Mr. [name parolee] cannot always be relied upon with appointments, but I also think a lot is expected from him. He was in [prison] for a long time and right now, what he needs is an encouraging approach but the focus currently seems to be merely on keeping his appointments. (Note from parole file Martin)

[Parolee] wanted to extend his curfew hours this weekend so he can take his girlfriend out for dinner. I gave him permission so he can practice with aspects of social life.(Note from parole file Pascal)

In sum, analyses of the case files of the participants yielded evidence of both a surveillance and a rehabilitative approach. On the one hand, a multitude of requirements characterised the conditional release of the current sample. Moreover, conditions indicated high levels of supervision intensity as a result of the nature of the offences combined with individual risk scores. On the other hand, notes from POs in the parole files suggested that they are understanding of the difficulty of meeting all these conditions and the trial-and-error nature of the desistance process. Violations of conditions were common, but often did not result in immediate revocation of release. Instead, alternative options were first deployed before official warnings were sent out.

### Experiences of Parole Supervision

The parole experience as reported by participants was not in all cases consistent with the impression from the parole files. Three months (T2) and a year after release (T3), the parole experience of (more than) half of the sample (*n* = 14 at T2 / *n* = 11 at T3) could be characterised as being primarily focused on surveillance, while for others (*n* = 9 at T2/*n* = 8 at T3), the supervision by the PO was perceived as engaged and supportive, resembling a caseworker approach. Most participants were consistent at both follow-up interviews in how they experienced their supervision (see Table 2). Only one participant shifted from experiencing his supervision as controlling in the first months after release to more focused on his desistance process at the 1 year after release follow-up. Furthermore, perceptions of parole supervision did not necessarily seem to be related to the co-occurrence of conditions, nor to desistance. Table 2 shows for example that the four men who had to comply with two conditions were equally likely to characterise their supervision as surveillance-oriented (or caseworker-oriented) as the seven men who had to fulfil six or more conditions.

A distinction was often made by the parolees between the requirements officially imposed by ‘the system’ (surveillance) and the human element of the supervision—the interaction with the PO (caseworker).

However, the human element in supervision did not automatically reflect a caseworker approach. In fact, only men who reported they felt their PO was supporting their journey in any way were assigned to the ‘caseworker-group’. When supervision according to the parolees predominantly entailed the monitoring of their compliance to the strict conditions and the PO was just doing his or her job, with no perceived extra effort in their view, the reported supervision style was classified as surveillance. In this sense, the PO was seen more or less as a pawn of the system, for example: ‘I thought they would be able to help you more instead of you just attending check-ins and explain what you have been doing’ (Sam, T3). Participants who experienced a surveillance approach were less positive about the parole experience although they usually described their PO as a nice person. They just did not find the supervision helpful in any way. For example, at the in-prison interview, Tony (CCC) who was involved in crime from a young age showed some insight into his deficits and his strengths when thinking of the future: ‘My dream is to open my own garage. I can fix any vehicle that needs to be fixed. I don’t have any papers, but I am creative. I know how to solve things and make money’ (T1). Then, 3 months after release, he said he had expected more from his parole supervision towards his goal, yet he thought his PO officer was a nice person:

JD: How is your parole officer?Tony: A *flex* chick, very relaxed and also honest.

JD: What is the role of parole supervision?Tony: they do not really help. She [PO] also says I have to wait with jobs, because I have to finish this aggression regulation course. She says, this is court ordered so we have to do it. […] I think it would be really good if there was some help with finding ways to get through the day. Even a project [unpaid] to have ex-prisoners sweeping the streets after a night out. Just to keep busy, you know. Parole supervision is more of an information desk: you come in with a question and they give you information, but help… No (T2).

At the final interview, Tony expressed frustration after a string of unsuccessful job interviews, including at garages:

Tony: They want people with diplomas. Yeah well, I don’t have one, but I am ten times more experienced than someone with papers. If I only got the chance to prove that…

JD: And the role of your PO?

Tony: She [PO] does arrange some things for me, if I can’t come to a check-in for example. But I feel the things she does are not helping me. [...] If you really want the best for me, please help me to gain some work experience. At least then I can put something new on my resume, something that’s real. (T3)

Tony’s expectations about parole supervision entailed strengths-focused building of capital, particularly in the area of employment. He was aware of skills he had, but at the same time lacked the opportunity to employ them and expected help from his PO, especially considering his own efforts to find a job. His disappointment with the experience of parole supervision (and thus the qualification of ‘surveillance-focused’ approach) may partly be explained by unrealistic expectations about what a PO can achieve and is within the remit of supervision.

The perception of supervision as a caseworker approach was characterised by the following: (a) the use of discretion, for example to adjust conditions in order to accommodate personal situation and tolerating missteps. This aspect of the PO resembles Lipsky’s [43] notion of the street-level bureaucrat, who is a mediator of official requirements; (b) a PO being perceived as going above and beyond official duties to help a parolee. Here, the PO is viewed as a social worker and advocate, assisting in rehabilitation efforts; and (c) a PO as being very supportive, listening, offering guidance and confronting bad behaviour. Here, the PO is experienced as a mentor. Reactions illustrating the caseworker style included ‘He supports me in every choice I make’ (Xavier, T2), ‘She already put in more hours than she officially was allowed to arrange everything asap’ (Casper, T3) and ‘He supports me, he helps me, he watches me’ (Oscar, T2).

### The Parole Experience and Dimensions of Desistance

In this section, it will be explored how supervisees navigated the release requirements and how this interacted with different dimensions of desistance. These dimensions of desistance are not mutually exclusive and are not necessarily ordered in a specific way. Thus, a person can report evidence of act-desistance and relational desistance; only identity desistance; or any other combination of dimensions (including, of course, no form of desistance at all). More than half of the sample (*n* = 14) were act-desisting up to a year after release and the majority (*n* = 16) mentioned some attempts at identity desistance in their interviews (even some men who were not act-desisting), for example as an employee, a parent, a loyal partner or a ‘good’ son. Half of the sample (*n* = 11) reported some form of recognition and/or support coming from either significant others, their PO or both. Finally, three men did not report on any of the dimensions at all. To clarify the ways in which the parole experience may help or hinder the desistance process as perceived by parolees, we analysed the reported experiences from pre-release up to a year after release in relation to the three dimensions of desistance.

### Act-Desistance

Two thirds of the men who reported a supervision experience resembling a caseworker approach were refraining from crime up to a year after release, while this was the case for half of the men who experienced supervision as surveillance-oriented (see Table 2 for offending across the three waves). The men who experienced a caseworker approach were generally positive about the (practical) help they received in their efforts to desist. The men who reported a supervision style that was classified as surveillance-oriented were less positive about their supervision experience, but nonetheless, for some of them, act-desistance seemed to be facilitated through constrained or instrumental compliance [9]. This compliance, then, was considered the best choice given rational calculations about the consequences of not adhering to the conditions (recall to prison). For example, Milo (CNN) made up his mind after release that he did not want to go back to prison and dealt with parole in the following way: ‘Whatever they say, I just say “ok”. I am not going to argue with them, they are my key to the outside world.’ (T3).

The use of electronic devices to monitor curfews and location bans created an externally imposed structure emphasising routine and self-control, placing physical restrictions on parolees’ conduct. The ankle bracelet was experienced as helpful in initial stages after release when it helped resistance of temptations, but became a hindrance when people were spending more months in society and were moving beyond this to establish new routines and relationships. For example, in prison, Sam (NNN) had mixed feelings about the upcoming tag (‘I get an ankle bracelet like a dog’ T1), but admitted to the positive effect it had on him 3 months after release when he was still unemployed:

It provides some structure. I don’t know what would have happened if I did not have the ankle bracelet. Maybe it would be the same, maybe not. (T2)

A year after release, Sam was full-time employed and reflected back on the past year of electronic monitoring:

I think I needed it back then. Especially then, when I did not have a job, yeah. You don’t do much all day. And inside your head, it’s chaotic, you are just out, your social welfare has not been arranged, you have to get used to outside. Then it’s a good thing you have a curfew and you have to be inside and all that. (T3)

However, he was still bound to the ankle bracelet a year after release and now felt it had lost its purpose and was nothing more than, literally, ‘a burden to his leg.’

Although half of the parolees who said their parole experience was surveillance-oriented refrained from offending up to a year after release, the other half continued to offend. For them, surveillance-oriented conditions did not facilitate constrained nor instrumental compliance. The conditions were also easily ‘cheated’ and could even mask the disengagement from the supervision for some individuals continuing crime. A few parolees who continued crime described in the interviews how they were able to fool their PO and the criminal justice system in thinking they were doing well and refraining from offending, when they were actually back committing crime instead (but no official offending on criminal records). One of them got caught by the end of the research period which then revealed his criminal activities to the criminal justice system and the PO, while the others managed to hide it from the officials throughout the follow-up period. Frequent check-ins as part of intensive supervision may then offer the illusion of control and surveillance, but may in fact fail to uncover a form of deceit (Irwin 1970) or ‘gameplaying’ [11], which refers to having little respect for regulation and seeking ways to fool the system. For example, three months after release Martin (ACC) said:

You have to give them that idea, or they are not going to leave you alone, you know what I mean? See, you have to step in here like you’re a whole other person otherwise you are not going to make it, man.JD: Otherwise they are going to pay a lot of attention to you?

Martin: Yes, a lot of attention. And you can tell them you want to work, but you also have to show it, you understand? So I go to a job interview and I really go, because they can verify it with GPS. And I take the business card along with me. I just give them the idea that I’m changed. (T2)

Martin’s quote suggests that his parolee performance (Irwin 1970) resembled the role of a reformed offender in the interactions with his PO; part of this role was a commitment to find a job.

### Identity Desistance

Many participants reported a real commitment to a worker identity, but also indicated that supervision requirements (mostly surveillance, but also rehabilitative) impeded identity desistance in several ways. First, on a practical level, it stood in the way of exercising new roles, particularly in the area of employment, but also the building of relationships, which became more of a concern (and frustration, due to the constraining conditions) after participants had been released for a few months. Dave (NNN) had to visit his PO weekly during working hours and expressed frustration and fear with regard to his newly acquired role of worker:

Every week, I have to take half a day off work to travel to [location of parole office] and report myself. Then it’s a 10 minute talk about how you are doing and what you are doing at the moment. I say ‘I work and everything is fine’ and I am dismissed. (T2)

Every week is the same. One time, I told her: no, I am not fine [...] I’m trying to get my life back in order, but you prevent me from doing so. You want me to come here [parole office], I have to leave my work for you. If I will be fired, don’t be surprised if I end up on the wrong path again. (T3)

Dave’s initial feelings with regard to supervision as being ‘processed’ [41] evolved into feelings of pessimism and fatalism when he was interviewed a year after release. The check-ins, psychological assessments and courses^12^ all took place during working hours, which in his perception continuously limited chances of full-time employment. In addition, location bans could block opportunities to re-establish social ties. Pascal’s location ban, for example, successfully removed him from his old criminal network but also made it difficult for him to invest in the relationship with his ill mother who lived in the restricted area. Although feeling hindered in ‘moving on’, both Dave and Pascal and many other men did not resort to crime.

Secondly, the use of electronic monitoring to enforce conditions such as curfews and location bans could be a constant reminder of the offender identity, instigating feelings of stigma. For example, Oscar (NNN) who was being subjected to the ankle bracelet for over a year after release talked about his constant awareness of ‘the system’ at the final interview:

Oscar: I know exactly what time I have to be inside and exactly when it has to be recharged, because they will call you within the next five minutes. The system is completely in my head.JD: Is it normal now?

Oscar: No, certainly not! I got used to it, but I don’t think it’s normal. Because of the ankle bracelet, I am not free. (T3)

Also, feelings of constantly having to prove oneself attacked the non-offender identity some men worked hard to maintain. Xavier (NNN) felt the mandatory drug tests he had to take became humiliating and lost their monitoring purpose a while after release:

After a while you think, is it really necessary? When in my case [after release], I didn’t smoke, I didn’t drink, in prison I didn’t smoke, I didn’t drink and now for over ten months you have seen I’m clean and it remains a constant. It is just a humiliation you know, constantly. (T3)

Although surveillance and rehabilitative parole conditions could be experienced as restrictive, sometimes the PO offered (practical) support further along the way and illustrated that a casework approach could be helpful in creating the necessary ‘space’ for clients to, aside from refraining from crime, also build their non-offender identity. For example, Pascal (CNN), who, in the pre-release interview, actually expected he would be involved in less serious crime after release, tried to adhere to the parole conditions and made attempts to live crime-free at the second and third interview. This was indeed a bumpy road as he had two major drug relapses after the first follow-up interview, but he confessed this immediately to his PO. Although it came as a surprise to his PO, she advised not to revoke his conditional release, because he had continuously showed motivation since release to set things straight.

The Probation Service thinks that sending him [Pascal] back to prison will not have the desired effect. This is because he is getting more and more insight into the motives of his drugs use and he is cooperative to be treated psychologically. (Note from parole file Pascal).

Pascal: I was really happy when I got another chance and did not have to go back to prison, it would be the same road when I would get out, so that’s not a solution. But I got an official warning and it really opened my eyes […] I’ve had some nightmares about the crime I was in for. I wanted to talk to a psychologist and she [PO] arranged that for me. (...) And fortunately, I could also enter the course [drug treatment] I had to finish in the evening. (T3)

In contrast to experiencing parole as solely surveillance-oriented the first months after release, Pascal mentioned it to resemble a caseworker approach a year after release because of his PO creating an opportunity for him to continue to build his non-offender identity and present his changed self. This was done by assisting him with the things he needed and downgrading his supervision to a less intensive level in order for him to work full-time. Thus, although strict supervision conditions could hinder attempts to fulfil new roles in the area of employment and relationships, POs could help to shape ways of achieving identity desistance when they actively assisted parolees’ efforts to change. This was more apparent in the one-year follow-up interviews than shortly after release.

### Relational Desistance

Receiving some recognition for attempts to go straight was reported by 11 out of 23 men. Three of these men, however, did not mention the role of the PO in supporting their desistance journey, but only their family and/or partner as a source of recognition. For the other eight men, the contribution of supervision to their relational desistance stood out as the most positive theme in their interviews.^13^ When POs affirmed parolees’ efforts to desist, they were seen as supportive and as mentors. Isaac (ANN) illustrated this view when he said he believed his supervision officer was an important factor in his attempts to stay straight when he slowly started to experience the ‘pain of goal failure’ [59]: ‘Every step I took [since release] was a red sign. No one hired me for my passion and capacities because of my past. (T3)’ Although Isaac had difficulties fulfilling the role of a good father and a worker (attempts at identity desistance), he still felt supported by his PO and managed to stay crime-free throughout the research period:

Isaac: She [PO] is the only person who believed in me. (..) She showed me she was not just a PO, but a person. And that’s what is [important] to me, you know. You have to be able to forget your job sometimes and just experience it together with this person.JD: What did she mean to you?Isaac: She gave me confidence not to do stupid things. Because I will make it on my own, but it’s hard to believe it yourself. You have a label, so relapsing is easy. Hanging in there is the hard part. And she motivated me ‘don’t blow it! Think about what you want and what you want is what you are going to do!’ […] She says that the way I think [about myself], that is how I have to present myself in life, so I can move on. (T3)

Isaac’s strong desire and movement towards desistance was recognised and appraised by his PO, which facilitated a process of pro-social labelling in line with the ‘looking-glass self’ as discussed by Marunaet al. [49]. Put differently, Isaac was able to view himself the way he believed his PO came to see him. She helped him gain confidence to maintain a crime-free life and verified the reformed pro-social identity of a non-offender at which he was working [38, 76]. The role of the PO in contributing to relational desistance seemed even more salient given the limited contact with other people after release. Some participants (Isaac, Pascal, Xavier, Simon, Casper, Oscar) were ‘knifing off’ old criminal networks, but due to strict curfews and location bans, possibilities to (re)connect with non-criminal others were scarce. Some participants (Casper, Pascal) even lacked or had limited contact with their family and in their case, the PO was the main contact with the world outside their home (see also [59]). As a result, chances for recognition and praise for their attempts at desistance were low.

## Discussion

Our analysis of longitudinal interview data with Dutch parolees combined with their parole files and criminal records provided a number of key findings. First, release conditions for men returning to society after prison predominantly involved risk management conditions such as curfews and location bans, enforced by electronic monitoring. However, importance was also given to addressing criminogenic needs, reflected in the fact that a majority of parolees were subjected to psychological assessment and treatment and/or placement in assisted living facilities. The parole files suggested that most parole officers employed a caseworker approach and seemed to be committed, engaged and making efforts to assist with rehabilitation. This finding supports the argued inconsistency between discourse and the everyday practice of criminal justice workers as documented by scholars in the USA and Europe [45, 57, 65]. Also among other criminal justice factors (such as correctional officers in prison), it has been found that their practice does not always match formal penal policy [15]. Furthermore, the way in which parole officers responded to violations was also suggestive of a rehabilitative approach, which resonates with recent comparative research carried out in several European countries concerning breach processes [8].

Secondly, parolees’ perceptions of their parole supervision did not necessarily correspond with the caseworker approach we had identified from the case files. Although the parole files suggested that most parole officers intended to employ a caseworker approach, half of the parolees perceived their officers’ supervision style as oriented more towards surveillance, aimed at monitoring them and making little effort to offer assistance (see also [60]). In some ways, what we described as a caseworker approach could be characterised as defiance of controls imposed ‘from above’. In the Netherlands, surveillance tasks for the supervising officers are described in much detail in manuals [6], but practice shows that POs have some ‘space’ (and creativity) in making decisions. This also illustrates the difficult position POs find themselves in handling discretionary power and balancing between supervision tasks in the context of the culture of control [25]. While we found some negative attitudes about the ability of POs to help, parolees were not as unequivocally negative as reported in earlier research with (ex-)prisoners in the USA [34]. These findings are in line with supervision literature describing these two approaches [17, 27, 29, 69, 74].

To understand how supervision perceptions were associated with efforts at desistance, it was helpful to distinguish between the three dimensions of desistance outlined by Nugent and Schinkel [59]: act-desistance (‘non-offending’), identity desistance (‘internalisation of a non-offending identity’) and relational desistance (‘recognition of change by others’). Strict supervision requirements appeared unsuccessful in preventing future criminal behaviour (act-desistance) for parolees who continued crime immediately after release. Ironically, this ‘illusion of control’ may lead to an underestimation of risks [71]. Nevertheless, some participants saw the benefits of electronic monitoring in the first chaotic months after release, for example through the provision of structure and guidance in daily activities. This finding is in line with some previous research done on electronically monitored individuals (as a stand-alone measure), which showed that curfew orders are linked to constraint-based compliance hereby reducing crime by ‘knifing off’ criminal networks and encouraging individuals to reconnect with pro-social bonds [35, 79]. However, strict supervision conditions such as check-ins, curfews and location bans were often felt to complicate efforts towards identity desistance by hindering chances of legitimate employment, (re)connecting with social ties and contributing to the experience of stigma, especially after the initial months after release. This could sometimes foster a pessimistic and fatalistic outlook, making desistance seem rather fragile (see also [30]).

Finally, the men who reported that their PO’s supervision style was caseworker-oriented tended to report more success with act, identity and relational desistance. Attempts at desistance tended to be supported when POs acted as mediators of requirements (similar to Lipsky’s street-level bureaucrat [43]), made serious and visible efforts to assist in rehabilitation goals and were supportive on a more emotional level. In line with previous research, these findings show that a positive relationship with the PO can have an important contribution to feelings of support and confidence in attempts at desistance, even when the parolee falls back into old behaviour.

A few limitations of the current study should be taken into consideration. First, the 1-year follow-up period of the current study is too short to gain an understanding of the perceived long-term impact of supervision. Processes of desistance take time to unfold and it could be possible that the meaning parolees give to supervision is better examined when they have had more time to reflect and think about life events [20]. In this context, it is known from previous research that the impact of the relationship between parole officer and parolee may not be experienced as substantial at first, but this can change over time—even after supervision has ended—when faced with life events and advice from the past suddenly seems to make sense [22]. Maybe parolees who described their supervision as surveillance-oriented, would in retrospect describe it as a caseworker approach after all. A second limitation is that no parole officers were interviewed about their supervision style; in order to get information about their perspective, we relied on (very detailed) notes, suggestions and remarks in the parole files. Thirdly, although our sample may be small, it overcomes some selectivity issues compared to samples used in previous research. And finally, in our analysis of the interviews, we focused on the parole experience and the different dimensions of desistance. It did not focus on contextual or demographic factors that could explain differences between the experiences and desistance process. For example, for individuals with strong social networks or legitimate ties to the community, the impact of supervision conditions and style may be quite different than for individuals who have less social or community support.

Future research could further explore the discrepancy in perceptions of ‘the system’ and of the people operating within this system, particularly the parole officers. Different scholars have pointed out that due to the shift from rehabilitation to risk management, the Dutch Probation Service has lost its original identity (of social workers) and now struggles to establish a new one balancing both tasks of surveillance and support [6, 78]. It could be the case that working on a pro-social identity is not only relevant for parolees but also for the Probation Service if they want to increase the likelihood of accumulating psychological legitimacy in the eyes of those subjected to their supervision [11].

McNeill [51] suggested to move towards a more desistance-focused supervision practice determined by the purpose of the intervention rather the offence precipitating it. The question arises what desistance-oriented supervision would look like. It has already been noted that, since desistance is a subjective and individualised process, parole should also be tailored as much as possible and pay attention to issues of identity [82]. The present study yields evidence that POs in the Netherlands who supervise prisoners returning to society after a relatively long imprisonment, for the most part already take such an individual approach instead of solely relying on risk principles. Despite the strict surveillance conditions, POs seem to make efforts at supporting supervisees to a crime-free life rather than on reporting them as soon as they violate one of these conditions. This seems in line with other research done on the impact of one-to-one interactions between supervising officers and the supervised individual, which illustrated the possible positive role of motivational and client-centred communications strategies [44, 80]. This longitudinal study suggests that a policy culture and discourse of risk management do not necessarily preclude desistance support in parole supervision in the Netherlands, due to discretionary power of parole officers. However, parole supervision in the Netherlands can possibly be more desistance-focused by working with, and/or discovering of, the strengths of the parolee [53]. One of the men in this study provided a compelling account of possibly ‘missed chances’ during supervision in recognising and extending his self-reported strengths. In this context, Lowenkamp et al. [44] point out that the highest risk individuals probably need much more of a rehabilitative approach than is provided by most supervision systems. So there might be considerable potential in this area to improve parole supervision. That said, even well-intended interventions can be experienced as burdensome and counterproductive [31, 57].

Yet, it does not suffice to have individual efforts from parolees and social support from significant others and supervisors; desistance also needs broader societal and political support in order to validate non-offender identities and full citizenship [21]. POs can be helpful in keeping parolees motivated to surmount their problems and serve as a catalyst to implement the desire to desist in their lives [32], yet structural opportunities remain scarce. For example, creating support among employers to offer a second chance for ex-prisoners on the labour market can be a worthwhile endeavour. For the men in this study who did eventually find employment, these ‘hooks for change’ [26] solidified their delicate non-offender identities and therefore seemed to support identity desistance. Although POs can serve as a social bridge, searching for suitable help or connecting the parolee to external agencies, the extent of their power to create opportunities remains limited. While POs may not be miracle workers, they can sometimes soften the impact of the bumpy road after release from prison.

## Electronic Supplementary Material

Supplementary Table 1 (DOCX 14 kb)

## Footnotes

1 In the Netherlands, the large majority of prisoners (93%) is released within a year after entering prison. The remaining 7% percentage is incarcerated for terms longer than 1 year [14].

2 McNeill [54] recently called this form ‘tertiary desistance’ in addition to primary desistance (non-offending) and secondary desistance (identity change).

3 According to the Conditional Release Act of 1 July 2008, prisoners who served two thirds of their imposed sentence are eligible for a conditional release under supervision with the general condition of not committing a new offence and additionally more specific, individually tailored conditions, such as drug tests and rehabilitative courses.

4 Sentence length was between 3 and 5 years.

5 In the Prison Project, the majority of participants had a short prison term so only four participants interviewed in the period November 2013–July 2014 were part of the original Prison Project and extra participants were recruited

6 For two respondents, we discovered that they were convicted for a sex offence after all, two respondents received another sentence while imprisoned, which meant they would not be released any time soon and therefore had to be excluded. One prisoner was detained for a shorter time than we initially thought and three prisoners were still in appeal.

7 Interview schedules can be obtained from the first author.

8 The letter classification (A, C, N) from the first interview wave concerning the pre-release expectations has been done by three researchers separately. There was high agreement and a few doubtful cases were discussed after which accordance was reached. The second letter classification (C, N) of the 3 months after release interviews was also done by three researchers and was solely based on the presence or absence of criminal behaviour. The last letter classification was done by the principal researcher, and was also solely based on the presence or absence of criminal behaviour.

9 Even if there was no official offending on the criminal records. There were no occurrences of official offending on the records which was not reported in the interviews.

10 It was difficult to establish a total number of violations, because violations were not always exactly counted and documented (as opposed to sanctions). More often, notes in parole files stated that the parolee missed ‘several’ check-ins or failed ‘multiple’ drug tests. We could, however, examine accurately how many parolees violated a specific conditions and if this was an incident or somewhat of a pattern (as opposed to knowing exactly how often).

11 A reprimand (written or verbal) is to make clear to the parolee that he reached a limit and to point out what the consequences can be if he continues this way. It is milder than an official warning; in the case of the latter, the Public Prosecution Service (OM) is also notified. In case of a serious violation, it is also possible to notify the PPS right away instead of giving an official warning.

12 Dave was assigned to live in an assisted living facility (see Table 2), but requested to live at his parents’ house instead. His request was approved and in turn he had to participate in a few courses.

13 Although most of these men did not have a partner, they did receive some support from family members aside from their PO.

## References

[1] Aebi MF, Chopin J (2018). Council of Europe Annual Penal Statistics SPACE II Survey 2016 Persons Serving Non-Custodial Sanctions and Measures in 2016.

[2] Aebi MF, Tiago MM, Burkhardt C (2016). SPACE I – Council of Europe Annual Penal Statistics: prison populations. Survey 2015.

[3] Bauwens A (2010). The use of method triangulation in probation research. European Journal of Probation.

[4] Beyens K, Persson A, Boone M, Maguire N (2018). Discretion and professionalism in a breach context. The enforcement of offender supervision in Europe.

[5] Blumstein A, Cohen J, Roth JA, Visher CA (1986). Criminal careers and “career criminals”.

[6] Boone M, Robinson G, McNeill F (2016). Community punishment in the Netherlands. A history of crises and incidents. Community punishment, European perspectives.

[7] Boone M, Beckmann M, Boone M, Maguire N (2017). Revocation and recall in the Netherlands. The enforcement of offender supervision in Europe.

[8] Boone MM, Maguire N (2017). The enforcement of offender supervision in Europe: understanding breach processes.

[9] Bottoms, A. (2001). Compliance and community penalties. In: A. Bottoms, L. Gelsthorpe & S. Rex (Eds.), Community penalties: Change and Challenges (pp. 87–116). London: Willan.

[10] Boyatzis RE (1998). Transforming qualitative information: thematic analysis and code development.

[11] Braithwaite, V. (2003). Dancing with tax authorities: motivational postures and non-compliant actions. *Taxing Democracy*, 15–39.

[12] Braun V, Clarke V (2006). Using thematic analysis in psychology. Qualitative Research in Psychology.

[13] Bushway SD, Piquero AR, Broidy LM, Cauffman E, Mazerolle P (2001). An empirical framework for studying desistance as a process. Criminology.

[14] De Looff J, Van de Haar M, Valstar H, Van Gemmert N (2017). DJI in getal 2012-2016. [Dutch prison statistics 2012-2016].

[15] Dirkzwager AJ, Kruttschnitt C (2012). Prisoners’ perceptions of correctional officers’ behavior in English and Dutch prisons. Journal of Criminal Justice.

[16] Dirkzwager, A. J. E., Nieuwbeerta, P., Beijersbergen, K. A., Bosma, A. Q., de Cuyper, R., Doekhie, J., et al. (2018). Cohort profile: the prison project—a study of criminal behavior and life circumstances before, during, and after imprisonment in the Netherlands. *Journal of Developmental and Life-Course Criminology*, 1–16.

[17] Ditton, J. & Ford, R. (1994). The reality of probation: a formal ethnography of process and practice, Adershot: Avebury*.*

[18] Doekhie J, Dirkzwager A, Nieuwbeerta P (2017). Early attempts at desistance from crime: prisoners’ prerelease expectations and their postrelease criminal behavior. Journal of Offender Rehabilitation.

[19] Durnescu I (2011). Pains of probation: effective practice and human rights. International Journal of Offender Therapy and Comparative Criminology.

[20] Farrall S, Calverley A (2006). Understanding desistance from crime. Theoretical directions in resettlement and rehabilitation.

[21] Farrall S, Bottoms A, Shapland J (2010). Social structures and desistance from crime. European Journal of Criminology.

[22] Farrall S, Hunter B, Sharpe GH, Calverley A (2014). Criminal careers in transition: the social context of desistance from crime.

[23] Feeley MM, Simon J (1992). The new penology: notes on the emerging strategy of corrections and its implications. Criminology.

[24] Flight S, Nauta O, Terpstra J (2011). Voorwaardelijk vrij. Evaluatie van de Wet voorwaardelijke invrijheidstelling.

[25] Garland D (2001). The culture of control.

[26] Giordano PC, Cernkovich SA, Rudolph JL (2002). Gender, crime and desistance: toward a theory of cognitive transformation. American Journal of Sociology.

[27] Glaser D (1964). The effectiveness of a prison and parole system.

[28] Gunnison E, Helfgott JB (2013). Offender reentry: beyond crime and punishment.

[29] Haggerty KD, Ericson RV (2006). The new politics of surveillance and visibility.

[30] Halsey, M., Armstrong, R., & Wright, S. (2016). “F*ck it!”: Matza and the mood of fatalism in the desistance process. *British Journal of Criminology*, azw041. 10.1093/bjc/azw041.

[31] Hayes D (2015). The impact of supervision on the pains of community penalties in England and Wales: an exploratory study. European Journal of Probation.

[32] Healy D (2012). Advise, assist and befriend: can probation supervision support desistance?. Social Policy & Administration.

[33] Healy D, O’Donnell I (2008). Calling time on crime: motivation, generativity and agency in Irish probationers. Probation Journal.

[34] Helfgott J (1997). Ex-offender needs versus community opportunity in Seattle, Washington. Federal Probation.

[35] Hucklesby A (2008). Vehicles of desistance? The impact of electronically monitored curfew orders. Criminology & Criminal Justice.

[36] Irwin, J. (1970). *The Felon*. Englewood Cliffs, NJ: Prentice-Hall.

[37] Kaeble, D., & Cowhig, M. (2018). *Correctional populations in the United States, 2016.* Department of Justice Bureau of Justice Statistics*.*

[38] King S (2013). Early desistance narratives: a qualitative analysis of probationers’ transitions towards desistance. Punishment & Society.

[39] Laub J, Sampson R (2003). Shared beginnings, divergent lives.

[40] LeBel TP, Burnett R, Maruna S, Bushway S (2008). The ‘chicken and egg’ of subjective and social factors in desistance from crime. European Journal of Criminology.

[41] Leibrich J (1993). Straight to the point: angles on giving up crime.

[42] Liem M (2016). After life imprisonment: reentry in the era of mass incarceration.

[43] Lipsky, M. (1980). *Street-level bureaucracy: Dilemmas of the individual in public services*. New York: Russell Sage Foundation.

[44] Lowenkamp CT, Holsinger A, Robinson CR, Alexander M (2014). Diminishing or durable treatment effects of STARR? A research note on 24-month re-arrest rates. Journal of Crime and Justice.

[45] Lynch M (2000). Rehabilitation as rhetoric: the idea of reformation in contemporary parole discourse and practice. Punishment and Society.

[46] Lyon D (1994). The electronic eye: the rise of the surveillance society.

[47] Maguire M, Peroud B, Raynor P (1996). Automatic conditional release: the first two years. Home Office Research Study 156.

[48] Maruna S (2001). Making good.

[49] Maruna S, Lebel TP, Mitchell N, Naples M (2004). Pygmalion in the reintegration process: desistance from crime through the looking glass. Psychology, Crime & Law.

[50] McCahill M, Finn RL (2012). The surveillance of ‘prolific’ offenders: beyond ‘docile bodies’. Punishment & Society.

[51] McNeill F, Chui WH, Nellis M (2003). Desistance-focused probation practice. Moving probation forward: evidence, arguments and practice.

[52] McNeill, F. (2009). *Towards effective practice in offender supervision*. Retrieved on (15 september 2017) from http://www.sccjr.ac.uk/news/SCCJR-Report-Released-Towards-Effective-Practice-in-Offender-Supervision/167.

[53] McNeill F, Shapland J, Farrall S, Bottoms A (2016). The fuel in the tank or the hole in the boat? Can sanctions support desistance. Global perspectives on desistance: rethinking what we know and looking to the future.

[54] McNeill F, Croall H, Mooney G, Munro R (2016). Desistance and criminal justice in Scotland. Crime, justice and society in Scotland.

[55] McNeill F, Beyens K (2013). Introduction: studying mass supervision. In: Offender supervision in Europe.

[56] McNeill F, Weaver B (2010). Desistance research and offender management. Review for the Ministry of Justice.

[57] McNeill F, Burns N, Halliday S, Hutton N, Tata C (2009). Risk, responsibility and reconfiguration: penal adaptation and misadaptation. Punishment & Society.

[58] Munn M (2011). Living in the aftermath: the impact of lengthy incarceration on post-carceral success. Howard Journal of Criminal Justice.

[59] Nugent B, Schinkel M (2016). The pains of desistance. Criminology & Criminal Justice.

[60] Opsal T (2009). Women on parole: understanding the impact of surveillance. Women & Criminal Justice.

[61] Opsal T (2012). ‘Livin’ on the straights’: identity, desistance, and work among women post-incarceration. Sociological Inquiry.

[62] Paternoster R, Bushway S (2009). Desistance and the feared self: toward an identity theory of criminal desistance. Journal of Criminal Law and Criminology.

[63] Payne BK, Gainey RR (1998). A qualitative assessment of the pains experienced on electronic monitoring. International Journal of Offender Therapy and Comparative Criminology.

[64] Petersilia J (2003). When prisoners come home: parole and prisoner reentry.

[65] Phelps MS (2011). Rehabilitation in the punitive era: the gap between rhetoric and reality in U.S. prison programs. Law & Society Review.

[66] Plaisier, J., & Pennekamp, S. (2009). *Planevaluatie reclasseringstoezicht*. Amsterdam: Impact R&D, WODC.

[67] Public Prosecution Service (Openbaar Ministerie) (2017). Centrale Voorziening voorwaardelijke invrijheidstelling (CVvi). Retrieved on September 5th 2018 from: https://www.om.nl/organisatie/ressortsparket-0/centrale-voorziening/.

[68] Rex S (1999). Desistance from offending: experiences of probation. Howard Journal.

[69] Rhine EE (1997). Probation and parole supervision: in need of a new narrative. Corrections Quarterly.

[70] Ricks EP, Eno Louden J, Kennealy PJ (2016). Probation officer role emphases and use of risk assessment information before and after training. Behavioral Sciences & the Law.

[71] Robinson G, McNeill F (2008). Exploring the dynamics of compliance with community penalties. Theoretical Criminology.

[72] Sampson RJ, Laub JH (1993). Crime in the making: pathways and turning points through life.

[73] Schinkel M (2014). Being imprisoned: punishment, adaptation and desistance.

[74] Seiter RP (2002). Prisoner reentry and the role of parole officers. Federal Probation.

[75] Shapland, J., & Bottoms, A. (2010). *Steps towards desistance: the potential role of criminal justice support*. Paper to the European Society of Criminology conference, Liege, September 2010.

[76] Stone, R., Morash, M., Goodson, M., Smith, S., & Cobbina, J. (2016). Women on parole, identity processes, and primary desistance. Feminist Criminology, 1557085116670004.

[77] Turnbull S, Hannah-Moffat K (2009). Under these conditions: gender, parole and the governance of reintegration. British Journal of Criminology.

[78] Van der Laan P (2017). Reclassering, beeldvorming en identiteit. PROCES.

[79] Vanhaelemeesch D, Vander Beken T, Vandevelde S (2014). Punishment at home: offenders’ experiences with electronic monitoring. European Journal of Criminology.

[80] Viglione J, Rudes DS, Taxman FS (2017). Probation officer use of client-centered communication strategies in adult probation settings. Journal of Offender Rehabilitation.

[81] Ward, T., & Maruna, S. (2007). *Rehabilitation: beyond the risk paradigm*. London: Routledge.

[82] Weaver B, McNeill F (2010). Travelling hopefully: desistance theory and probation practice.

[83] Yahner J, Visher C, Solomon A (2008). Returning home to parole: former prisoners’ experiences in Illinois, Ohio, and Texas.

