# Supplementary material for: Managing Risk or Supporting Desistance? A Longitudinal Study on the Nature and Perceptions of Parole Supervision in the Netherlands
Source: J Dev Life Course Criminol. 2018 Dec 5;4(4):491–515. doi: 10.1007/s40865-018-0097-6 (PMC6390716; doi:10.1007/s40865-018-0097-6)
Supplement: Supplementary file 1 — (DOCX 14 kb) [file 40865_2018_97_MOESM1_ESM.docx]

Table 1

*Perceptions of supervision in relation to three aspects of desistance.*

|  | Experienced supervision style | |
| --- | --- | --- |
|  | Caseworker  n=10 | Surveillance  n=13 |
| Act desistance | 8 (80%) | 6 (46%) |
| Identity desistance | 9 (90%) | 7 (54%) |
| Relational desistance | 6 (60%) | 5 (38%) |
| No form of desistance | 0 (0%) | 3 (23%) |
